# LINC01128 facilitates the progression of pancreatic cancer through up-regulation of LDHA by targeting miR-561-5p

**DOI:** 10.1186/s12935-022-02490-5

**Published:** 2022-02-22

**Authors:** Min Zhong, Zhi Fang, Bin Ruan, Jianping Xiong, Junhe Li, Zhiwang Song

**Affiliations:** 1 Department of Oncology, The First Affiliated Hospital of Nanchang University, 17 Yongwai Street, Nanchang, 330006 Jiangxi China; 2 Department of Jiangxi Key Laboratory for Individualized Cancer Therapy, 17 Yongwai Street, Nanchang, 330006 Jiangxi China

**Keywords:** LINC01128, miR-561-5p, Lactate dehydrogenase A, Pancreatic cancer, Proliferation, Invasion

## Abstract

**Background:**

Long non-coding RNAs (lncRNAs) regulate tumor development and metastasis in several types of cancers through various molecular mechanisms. However, the biological role of most lncRNAs in pancreatic cancer (PC) remains unclear. Here, we explored the expression, biological functions, and molecular mechanism of LINC01128 in PC.

**Methods:**

Quantitive reverse transcription PCR was used to detect the expression level of LINC01128 in PC tissues and different PC cell lines. A loss-of-function and gain-of-function experiment was used to explore the biological effects of LINC01128 on PC carcinogenesis in vitro and in vivo. Western blot analysis, subcellular fractionation experiment, luciferase reporter gene assay, and MS2-RNA immunoprecipitation experiment were used to study the potential molecular mechanism of LINC01128 during carcinogenesis.

**Results:**

The expression of LINC01128 was upregulated in PC tissues and cell lines, and overexpression of LINC01128 was significantly related to the poor prognosis of patients with PC. Furthermore, silencing LINC01128 significantly inhibited the proliferation, migration, invasion, and epithelial-mesenchymal transition (EMT) of PC cells in vitro and tumor growth in vivo, while LINC01128 overexpression promoted these processes. Further research showed that LINC01128 acted as a sponge for microRNA miR-561-5p, and lactate dehydrogenase A (LDHA) was the downstream target gene of miR-561-5p. It was also revealed that the expression of miR-561-5p in PC was decreased, and a negative correlation between miR-561-5p and LINC01128 was revealed. Based on rescue experiments, LDHA overexpression partially restored the inhibitory effect of LINC01128 knockdown on proliferation, migration, and invasion of PC cells.

**Conclusions:**

LINC01128 promotes the proliferation, migration, invasion, and EMT of PC by regulating the miR-561-5p/LDHA axis, suggesting LINC01128 may be a new prognostic marker and therapeutic target in PC.

**Supplementary Information:**

The online version contains supplementary material available at 10.1186/s12935-022-02490-5.

## Introduction

Pancreatic cancer (PC) is one of the most common malignant tumors, with 458,918 new cases and 432,242 deaths worldwide in 2018 [1]. Although the diagnosis and treatment of PC have been improved in recent years, the efficacy of surgery and chemotherapy is still unsatisfactory, with a 5-year survival rate of only 5% [2, 3]. Therefore, it is urgent to explore novel molecular mechanisms of PC, identify new prognostic biomarkers, and develop more effective treatment strategies.

Long non-coding RNAs (lncRNAs) are transcripts greater than 200 nucleotides in length; they play an important role in many biological processes, including cell-cycle regulation, lineage differentiation, stem cell pluripotency, and cancer progression [4]. Recent studies have shown that lncRNAs are aberrantly expressed in plenty of cancers, acting as oncogenes, tumor suppressors, or both, which participates in various biological processes, such as proliferation, migration, and metastasis [5–7]. Several lncRNAs have been reported to be related to the growth, invasion, and metastasis of PC [5, 8, 9], however, their specific roles and mechanisms in the occurrence of PC have not yet been elucidated.

LINC01128, located on chromosome 1 (827,591–859,446), is a novel lncRNA identified in cancer. Previous studies have shown that LINC01128 can enhance cell proliferation, migration, and invasion by upregulating stratifin (SFN) expression after binding with microRNA (miRNA) miR-383-5p [10]. In contrast, another study demonstrated that LINC01128 acted as a tumor suppressor in acute myeloid leukemia [11]. In our current study, we found LINC01128 was upregulated in PC tissues and cell lines, and loss-of-function and gain-of-function experiments showed that LINC01128 promoted cell proliferation, migration, invasion, and epithelial-mesenchymal transition (EMT) in vitro and tumor growth in vivo. Mechanistically, LINC01128 acted as a competing endogenous RNA (ceRNA) that upregulated the protein expression of lactate dehydrogenase A (LDHA) by sponging miR-561-5p. In summary, our study reveals a novel LINC01128/miR-561-5p/LDHA pathway in PC, indicating that LINC01128 may serve as a potential prognostic marker for the occurrence and development of PC, thereby providing a new target for PC treatment.

## Methods

### Clinical samples

48-primary PC specimens and their adjacent tissues were obtained from the First Affiliated Hospital of Nanchang University with the written consent of the patient. None of the patients received chemotherapy or radiotherapy prior to surgery. The study was approved by the Human Research Ethics Committee of the First Affiliated Hospital of Nanchang University.

### Cell lines and cell culture

Human PC cell lines (SW1990, AsPC-1, PANC-1, BxPC-3, Capan-2, and MIAPaCa-2) and a normal pancreatic epithelial cell line (HPDE) were purchased from the American Type Culture Collection (ATCC). All cells were cultured in Dulbecco’s modified Eagle’s medium (DMEM; Gibco, USA) with 10% fetal bovine serum (Invitrogen, USA) in an incubator under 5% CO_2_ at 37 °C. All cell lines were tested for mycoplasma contamination and authenticated using short tandem repeat (STR) profiling.

### Real-time quantitative PCR (qRT-PCR)

First, TRIzol (Invitrogen) was used to extract total RNA from fresh pancreatic tissue samples and PC cells. Easy Script cDNA Synthesis Kit (TransGen Biotech) was used to reverse transcribed the extracted RNA into complementary DNA (cDNA). Subsequently, the Step One Plus real-time PCR system (Applied Biosystems) and Fast Start Universal SYBR Green Master Mix (Takara) were used for qRT-PCR detection. Table 1 presented the primer sequences used in our manuscript. The relative expression level was analyzed via using 2^−ΔΔCT^ method.

**Table 1:** Primer list

Gene	Forward primer	Reverse primer
LINC01128	AAGGTGAGGTGAGAGGACAGGAAG	CAAGGCAGGCACTCAACGGTAG
miR-561	CAAAGUUUAAGAUCCUUGAAGU	TCAACTGGTGTCGTGGAGTCGGC
LDHA	GAGTGGAATGAATGTTGCTGGTGTC	CCAGGATGTGTAGCCTTTGAGTTTG
GAPDH	CCAGCCGAGCCACATCGCTC	ATGAGCCCCAGCCTTCTCCAT
U6	CTCGCTTCGGCAGCACATATACT	ACGCTTCACGAATTTGCGTGTC

LINC01128, long intergenic non-protein coding RNA 1128; LDHA, lactate dehydrogenase A; GAPDH, glyceraldehyde 3-phosphate dehydrogenase

### Cell transfection

LINC01128 overexpression plasmid, short hairpin RNA (shRNA) against LINC01128, LDHA overexpression plasmid, LDHA inhibitors, miRNA mimics, and miRNA inhibitors were purchased from Gene Pharma (Shanghai, China). The cells under a density of 50–70% were transfected with the corresponding plasmid via Lipofectamine 2000 (Invitrogen; Thermo Fisher Scientific, Inc.) according to the manufacturers’ guidance.

### Cell proliferation and colony formation assays

A density of 2000 cells per well and 500 cells each well were needed for Cell Counting Kit 8 (CCK-8) assay and colony formation assays after 48 h-transfection, respectively. The details for each experiment could be seen as previously described [12].

### Migration and invasion assays

Cells at a density of 3 × 10^4^ were seeded into the upper transwell chamber (BD Biosciences, USA) under FBS-free medium condition with or without Matrigel (Corning, USA), while the lower chamber was filled with complete medium. The chamber was collected and stained with crystal violet containing 1% methanol after 24-h incubation.

### Luciferase reporter assay

An online software site named StarBase3.0 (http://starbase.sysu.edu.cn/) was used in our study to predict the binding sites among LINC01128, miRNA-561 and LDHA [13]. Lipofectamine 3000 transfection reagent was further used to transfect luciferase vector (Promega, USA) with wild-type LINC01128 or mutant-type LINC01128 into PC cells with the mimic. The luciferase activity of the reporter plasmid was finally detected via the double luciferase reporter gene assay system (Promega, USA).

### Subcellular fractionation assay

APARIS kit purchased from Life Technology Company, USA was used to isolate nuclear and cytoplasmic RNA, which can be referred to the manufacturer's instructions for details. The levels of β-actin, U6, and LINC01128 of each fraction were subsequently analyzed and evaluated via qRT-PCR.

### MS2-RIP assay

We co-transfected SW1990 and BxPC-3 cells with pcDNA-MS2, pcDNA-MS2-LINC01128, or pcDNA-MS2-LINC01128-MUT (miR-561-5p), along with pMS2-GFP (Addgene), using Viafect reagent. After 48 h, GFP antibody (Roche) and the Magna RIP™ RNA-Binding Protein Immunoprecipitation Kit (Millipore, Bedford, MA, USA) were used to collect and lyse the cells and perform RIP analysis according to the manufacturer's instructions. Finally, purified RNA was measured using qRT-PCR to confirm the presence of the bound target.

### Western blot

Western blotting was performed as described previously [12]. Antibodies against β-actin, E-cadherin, Slug, Snail, N-cadherin, vimentin, and LDHA were purchased from Cell Signaling Technology. Quantitative analysis of protein expression was realized by ImageJ software (National Institutes of Health, Bethesda, MD).

### In vivo analysis

A total of 18 five-week-old female nude mice purchased from the National Laboratory Animal Center (Beijing, China) were randomly assigned into three groups for the following experiments. The cells about 1 × 10^6^ from the negative control (NC) group, the LINC01128 overexpression group and the LINC01228 knockdown group were separately subcutaneously injected into the right axilla of the corresponding group of nude mice. Tumor volume was measured every four days using the formula: length × width^2^ × 0.5. After 28-day feed, the nude mice were killed by cervical dislocation under ether anesthesia and the tumors were collected and recorded. All animal experiments were ethically supported by the Animal Research Ethics Committee of Nanchang University.

### Statistical analysis

SPSS software (version 25.0) was used for statistical analysis. Student's t-test and one-way ANOVA were used to evaluate the statistical significance of comparisons between two groups and more than two groups, respectively. Spearman’s correlation analysis was performed using MATLAB. Survival plots were drawn according to the Kaplan–Meier analysis. All experiments were repeated at least three times. p < 0.05 was considered statistically significant.

## Results

### The up-expression of LINC01128 in PC tissues and cell lines is related to a poor prognosis in PC patients

**Fig. 1:**
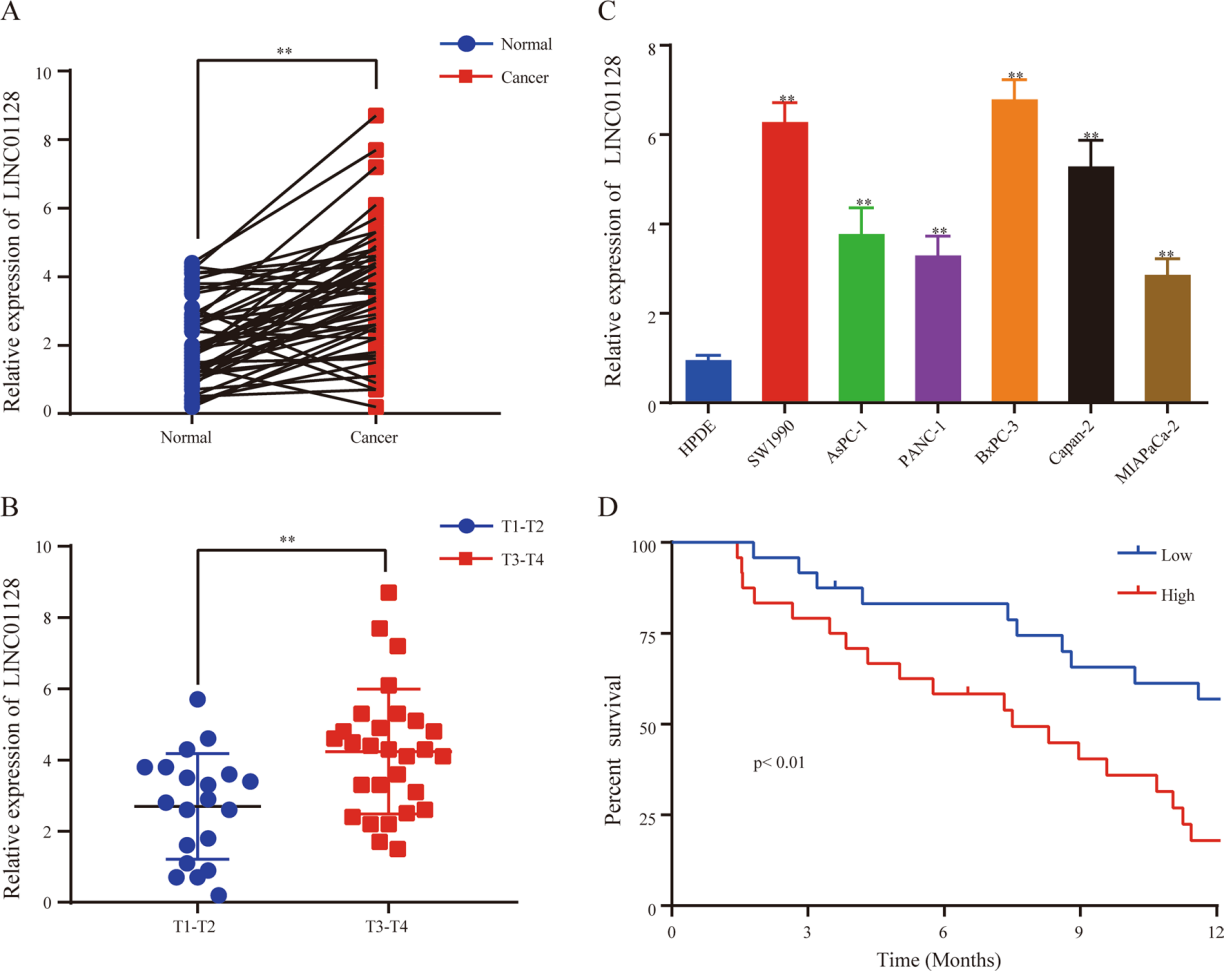
LINC01128 was upregulated in pancreatic cancer (PC) tissues and cell lines and associated with poor prognosis in patients with PC. **a**–**c** The expression level of LINC01128 in PC tissues and adjacent normal tissues, normal pancreatic epithelial cell lines, and PC cell lines was detected using qRT-PCR. **d** Kaplan–Meier analysis was used to evaluate the relationship between the expression level of LINC01128 and the overall survival of patients with PC. Data are expressed as the mean ± SD of three independent experiments. *p < 0.05, **p < 0.01

To determine the exact role of LINC01128 in the carcinogenesis of PC, qRT-PCR was used to detect the expression levels of LINC01128 in 48 pairs of PCs and their adjacent normal tissues. LINC01128 expression in adjacent normal tissues was significantly lower than in PC tissues (Fig. 1a). Furthermore, the upregulated expression of LINC01128 was significantly associated with clinical stage (Fig. 1b). In addition, compared to the normal pancreatic HPDE cells, LINC01128 was highly expressed in PC cell lines (Fig. 1c). Additionally, the clinical follow-up data of the enrolled patients with PC showed that patients with higher LINC01128 expression had shorter overall survival than patients with lower LINC01128 expression (Fig. 1d). In summary, these findings indicate that LINC01128 is upregulated in PC tissues and cell lines and is associated with poor prognosis in patients with PC.

### LINC01128 knockdown inhibits the proliferation, migration, invasion, and EMT of PC and induces G2/M phase arrest

**Fig. 2:**
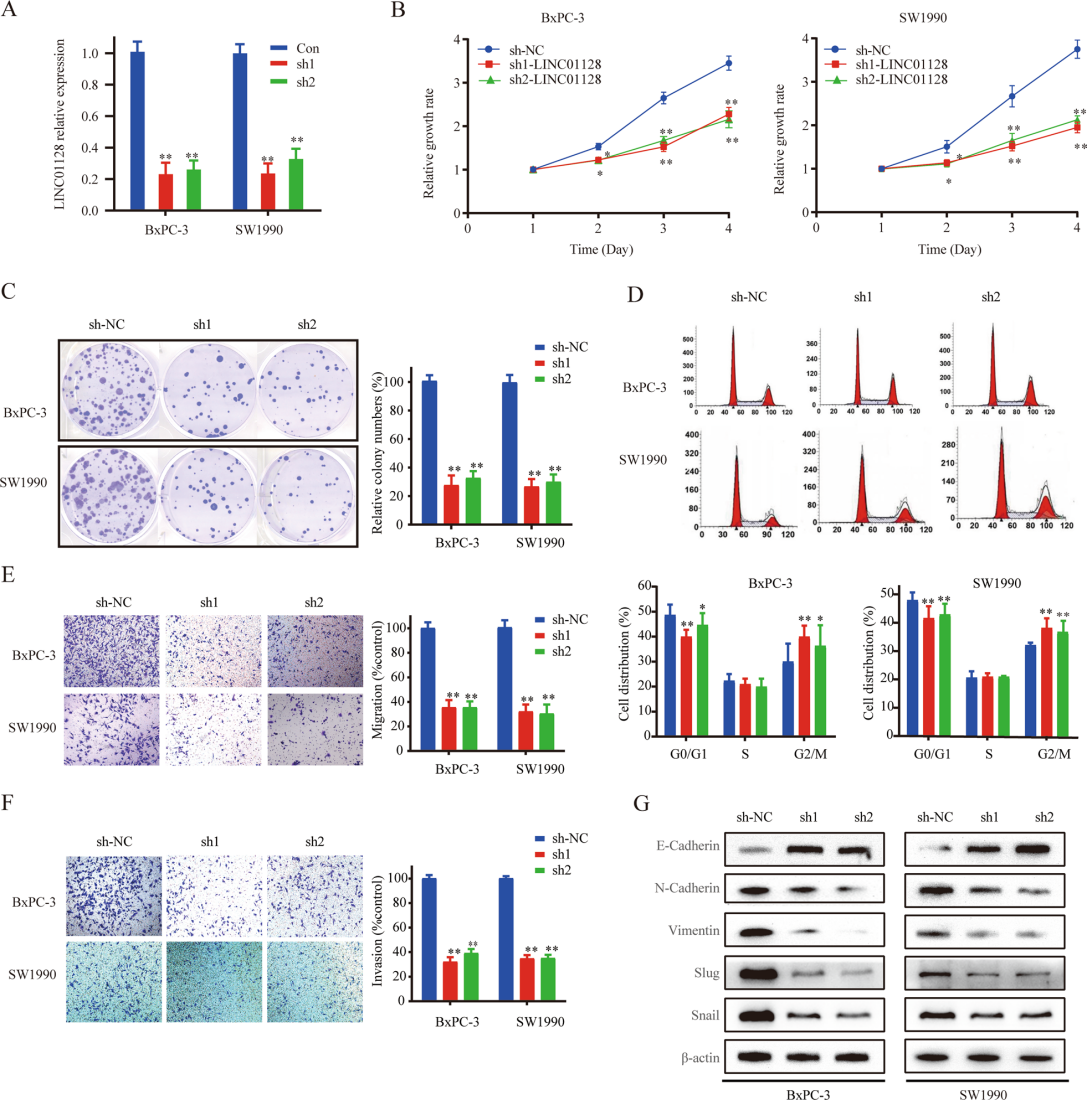
LINC01128 knockdown inhibited proliferation, migration, invasion, and EMT and induced G2/M phase arrest. **a** The transfection efficiency of SW1990 and BxPC-3 cells after LINC01128 knockdown by shRNA was analyzed using qRT-PCR. **b**, **c** CCK-8 assays and clone formation assays were used to determine the cell proliferation rate and cell viability. **d** Fluorescence-activated cell sorting (FACS) was used to analyze cell cycle distribution. **e**, **f** Cell migration and invasion abilities were analyzed using transwell migration and invasion assays. **g** The protein expression of E-ceaderin, N-cadherin, Slug, Snail and Vimentin was detected by western blot analysis. Data are presented as mean ± SD of three independent experiments. *p < 0.05, **p < 0.01

We next analyzed the effect of LINC01128 on PC cell proliferation. Considering the highest expression of LINC01128 in BxPC-3 and SW1990 cells exhibited in Fig. 1c, these two cells were chosen to further examine the biological effect LINC01128. Subsequently, a stably transfected cell line with LINC01128 knockdown (sh-LINC01128) and a negative control (sh-NC) cell line were established in SW1990 and BxPC-3 cells (Fig. 2a). CCK-8 and cell clone assays showed that the cell proliferation rate and colony-forming ability of the sh-LINC01128 group were significantly lower than in the sh-NC group (Fig. 2b, c). In addition, flow cytometry was used to analyze the role of LINC01128 in cell cycle regulation. Compared to that in the sh-NC group, the percentage of cells in the G2/M phase was significantly increased in the sh-LINC01128 group (Fig. 2d), indicating a role of LINC01128 in G2/M phase arrest. These results prove that LINC01128 knockdown is able to induce G2/M phase arrest, which may be the reason for the observed inhibition of proliferation.

Considering the role of LINC01128 from clinical samples in lymph node metastasis and vascular infiltration, a transwell assay was used to investigate the role of LINC01128 in migration and invasion. Compared to the sh-NC group, cell migration and invasion were obviously deceased in the sh-LINC01128 group (Fig. 2e, f). The transformation of tumor cells from epithelioid cells to mesenchymal cells with migration and invasion potential is called EMT, which plays a very important role in cancer progression [14]. Moreover, the epithelial marker E-cadherin was found to be down-regulated, the mesenchymal markers vimentin and N-cadherin were upregulated during EMT reversely [15]. Interestingly, we also confirmed that the decline of LINC01128 resulted in an upregulation of E-cadherin and a downregulation of N-cadherin, Vimentin, Slug, and Snail (Fig. 2g). The above results indicate that LINC01128 knockdown could inhibit migration, invasion, and EMT in PC.

### LINC01128 overexpression enhances proliferation, migration, invasion, and EMT

**Fig. 3:**
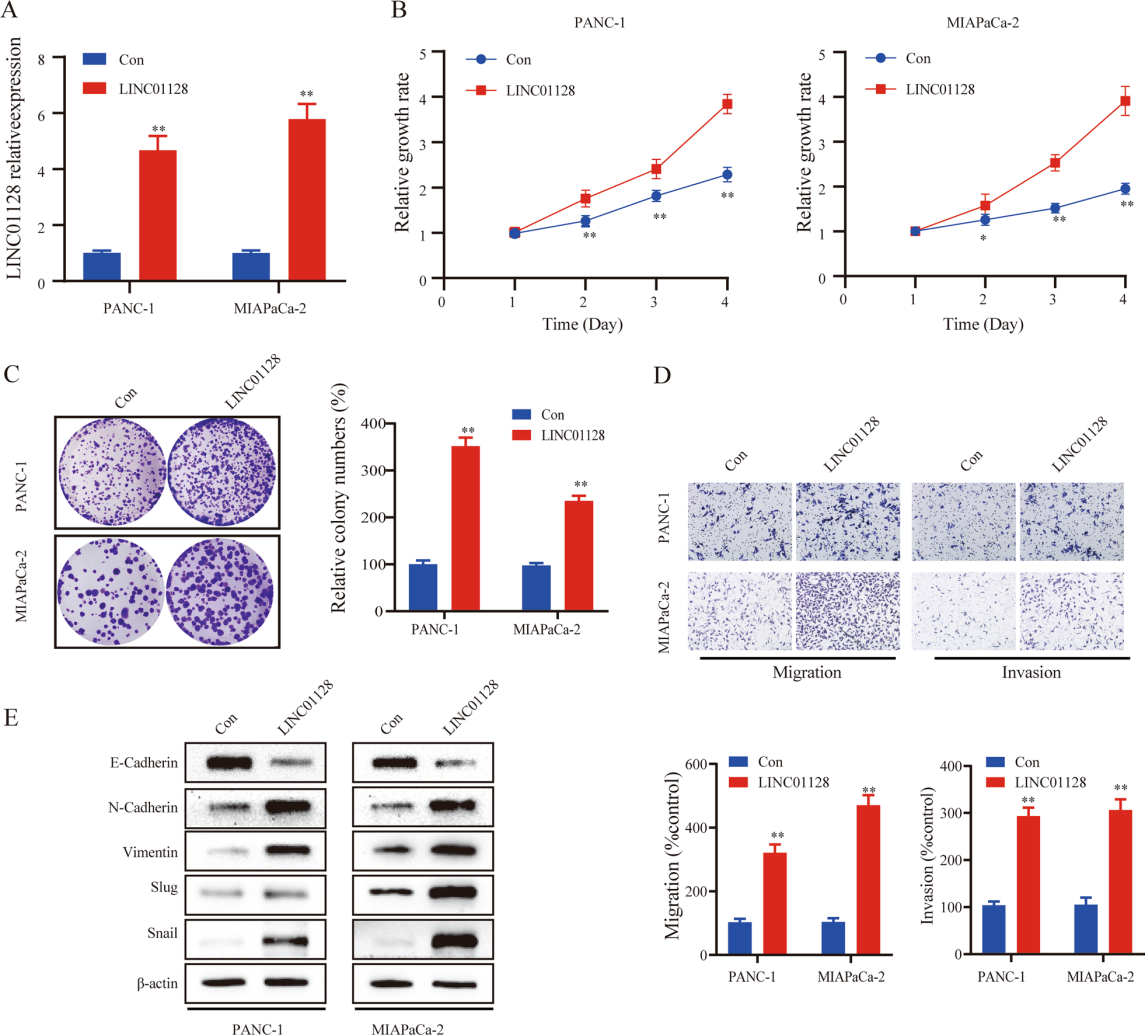
LINC01128 overexpression promoted proliferation, migration, invasion, and EMT in pancreatic cancer. **a** The transfection efficiency of PANC-1 and MIAPaCa-2 cells after LINC01128 overexpression was analyzed using qRT-PCR. **b**, **c** CCK-8 assays and clone formation assays were used to determine the cell proliferation rate and cell viability. **d** Cell migration and invasion abilities were analyzed using transwell assays. **e** Western blot analysis was conducted to evaluate the impact of LINC01128 on EMT progression. Data are presented as mean ± SD of three independent experiments. *p < 0.05, **p < 0.01

Stable LINC01128 overexpression and negative control cell lines were constructed in MIAPaCa-2 and PANC-1 cells (Fig. 3a). Cell clone assays and CCK-8 indicated the colony-forming ability and cell proliferation rate of the LINC01128 group were greatly higher than in the NC group (Fig. 3b, c). In addition, cell migration and invasion in the LINC01128 group were significantly increased compared to the NC group (Fig. 3d). Finally, western blot analysis confirmed that LINC01128 overexpression reduced the E-cadherin expression but increased the expressions of N-cadherin, vimentin, Slug, and Snail (Fig. 3e). In summary, these results indicate that the overexpression of LINC01128 enhances cell proliferation, migration, invasion, and EMT in PC.

### LINC01128 functions as a molecular sponge of miR-561-5p

**Fig. 4:**
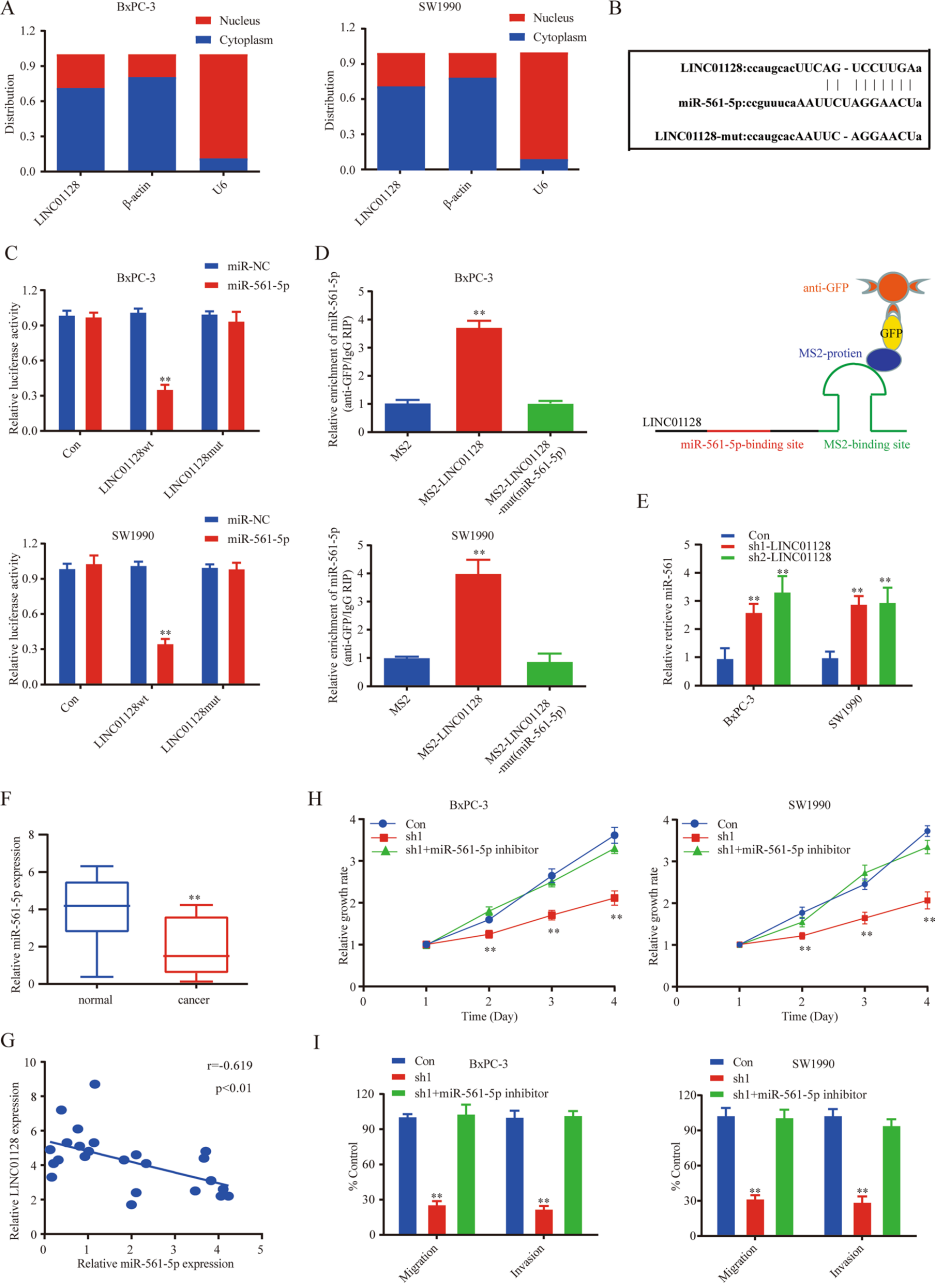
LINC01128 was a sponge for miR-561-5p. **a** The subcellular location of LINC01128 was determined by subcellular fractionation assay. **b** Sequences of WT-LINC01128, Mut-LINC01128, and miR-561-5p. **c** Luciferase assay of SW1990 and BxPC-3 cells transfected with LINC01128-MUT or LINC01128-WT reporter gene, together with NC or miR-561-5p. **d** MS2-RIP was applied to evaluate endogenous miR-561-5p associated with the MS2-tagged LINC01128. **e** The expression level of miR-561-5p in BxPC-3 and SW1990 cells. **f** The expression level of miR-561-5p in PC tissues and adjacent normal tissues. **g** The correlation between LINC01128 and miR-561-5p levels was determined by Spearman's rank correlation test analysis. **h**, **i** CCK-8 assays and transwell assays were used to detect the proliferation, migration, and invasion ability. Data are presented as mean ± SD of three independent experiments. *p < 0.05, **p < 0.01

To explore the potential molecular mechanism of LINC01128 in PC carcinogenesis, we first explored the subcellular location of LINC01128. The results suggested that LINC01128 was mainly distributed in the cytoplasm (Fig. 4a), indicating LINC01128 may perform as a miRNA sponge. Next, we used StarBase3.0 to predict the potential downstream targets of LINC01128 and identified several miRNAs as candidates. Following to the fold change expression of twenty predicted miRNAs in SW1990 cells transfected with sh-LINC01128 (Table 2), we chose miR-561-5p as our candidate miRNA (Fold change = 3.37; p < 0.01). Binding site information of LINC01128 and miR-561-5p was shown in Fig. 4b. The luciferase reporter gene test results confirmed that miR-561-5p mimics significantly reduced the luciferase activity of WT-LINC01128, whereas the luciferase activity of MUT-LINC01128 did not change significantly (Fig. 4c). To further identify whether LINC01128 directly binds to endogenous miR-561-5p, we performed MS2-RNA immunoprecipitation (RIP) assays to pull down the endogenous miRNAs related to LINC01128. Compared to the empty and mutant plasmids, the wild-type LINC01128 vector tagged with MS2 was rich in miR-561-5p (Fig. 4d).

**Table 2:** Fold change expression of twenty predicted miRNAs in cells transfected with sh-LINC01128

miRNAs	Fold change	*p* value
miR-363-3p	2.49	< 0.01
miR-92b-3p	1.46	< 0.01
miR-92a-3p	2.41	< 0.01
miR-367-3p	2.35	< 0.01
miR-32-5p	1.49	< 0.01
miR-363-3p	1.31	< 0.01
miR-25-3p	1.55	< 0.01
miR-208a-3p	1.24	< 0.01
miR-519b-3p	1.09	< 0.01
**miR-561-5p**	**3.37**	**< 0.01**
miR-27b-3p	2.21	< 0.01
miR-599	1.13	< 0.01
miR-299-3p	1.71	< 0.01
miR-95-3p	1.44	< 0.01
miR-4662a-3p	2.03	< 0.01
miR-2278	2.21	< 0.01
miR-2115-3p	1.47	< 0.01
miR-137	1.15	< 0.01
miR-663a	1.91	< 0.01
miR-3679-5p	2.44	< 0.01

Bold values indicate among the 20 miRNAs predicted in SW1990 cells transfected with sh-LINC01128, the highest fold change was miR-561-5p

Furthermore, silencing of LINC01128 upregulated the level of miR-561-5p compared to the sh-NC group (Fig. 4e). To further evaluate the expression of miR-561-5p in tumor tissues, we preformed qRT-PCR. The results revealed that the miR-561-5p levels were lower in PC tissues than normal tissues, and the expression level of LINC01128 was negatively correlated with that of miR-561-5p (Fig. 4f, g). To further explore the exact biological effect of miR-561-5p, we carried out CCK-8 assay, colony formation assay and transwell assay with miR-561-5p mimics. Results showed that the cellular growth, proliferation, migration and invasion ability was decreased in miR-561-5p mimics compared to control group (Additional file 1: Fig. S1a–c). In addition, LINC01128 promoted the proliferation, migration, and invasion of PC cells at least by sponging miR-561-5p in part (Fig. 4h, i). In summary, LINC01128 can be used as a miR-561-5p molecular sponge.

### LDHA is a downstream target of miR-561-5p in PC

**Fig. 5:**
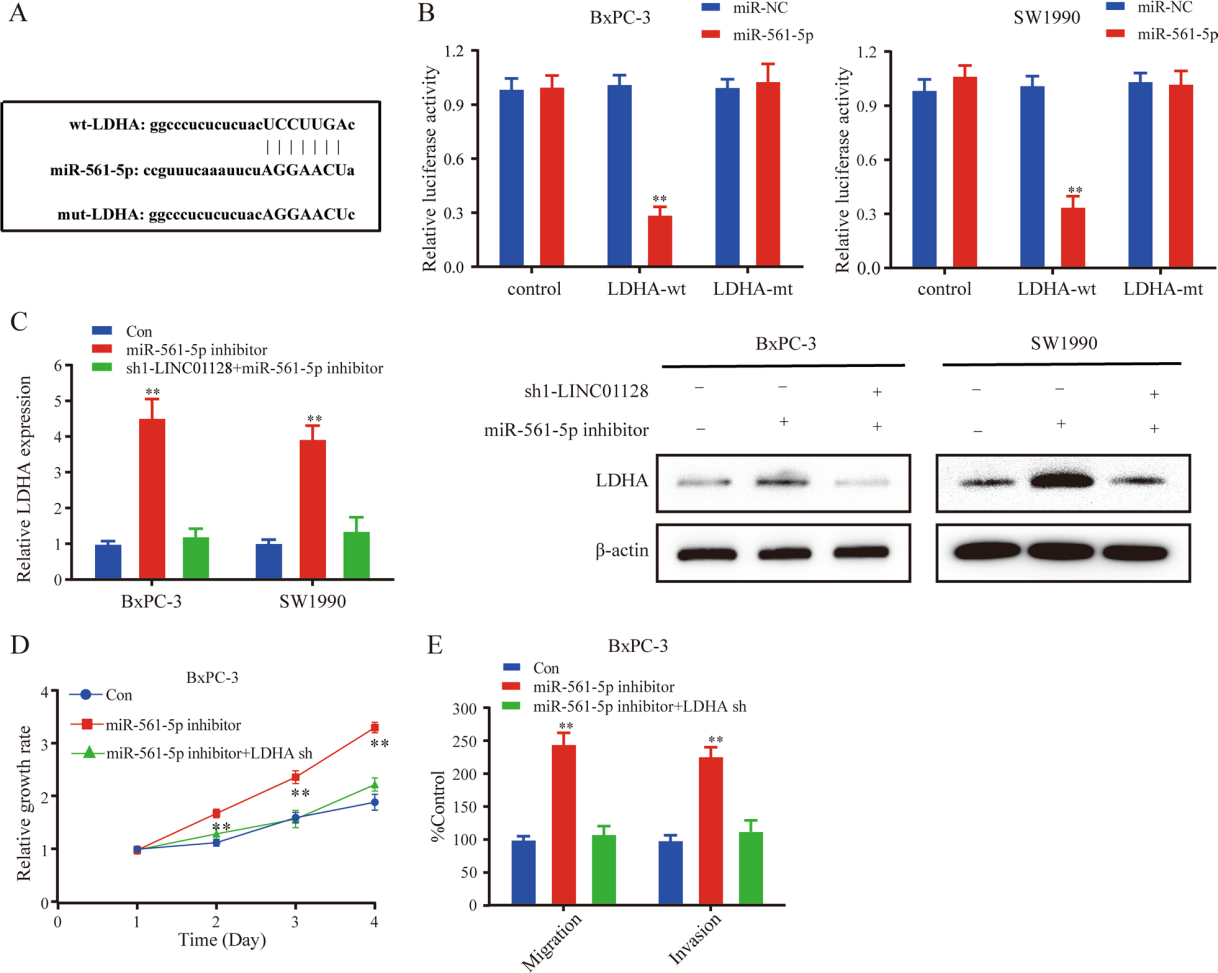
LINC01128 silencing suppressed tumor growth in pancreatic cancer cells and LDHA protein in vitro. **a** Schematic diagram of miR-561-5p, wild-type LDHA, and LDHA mutant. **b** The luciferase reporter assay showed luciferase vitality in the combination of miR-561-5p mimic/control and LDHA wild-type/mutant. **c** QRT-PCR and western blotting were performed to detect LDHA expression of LINC01128 silencing and co-transfecting LINC01128 shRNA and miR-561-5p at the transcription and translation levels. **d**, **e** CCK-8 assays and transwell assays were used to detect cell proliferation, migration, and invasion of BxPC-3 cells transfected with miR-561-5p inhibitor and co-transfected with miR-561-5p inhibitor and LDHA shRNA. Data are presented as mean ± SD of three independent experiments. *p < 0.05, **p < 0.01

In general, miRNAs perform post-transcriptional functions by base pairing with 3'-untranslated regions to inhibit protein synthesis [16]. LDHA was predicted as a potential downstream target of miR-561-5p by searching in StarBase3.0 (Fig. 5a). Subsequently, the luciferase reporter assay confirmed the hypothesis that LDHA was a direct target of miR-561-5p (Fig. 5b). First of all, the expression of LDHA was valuated after LINC01128 upregulation or downregulation. As shown in Additional file 2: Fig. S2a–d, suppressing LINC01128 inhibited both the mRNA expression and protein expression of LDHA while enhancing LINC01128 reversed the results. In addition, the expression of LDHA was upregulated by miR-561-5p inhibitors, however, co-transfection of sh-LINC01128 and miR-561-5p inhibitors reversed the effect of miR-561-5p knockdown on LDHA transcriptionally and translationally (Fig. 5c). So far, our results prove that LDHA is the direct target gene of miR-561-5p. The results of functional experiments further showed that LDHA silencing significantly inhibited the promotion of miR-561-5p inhibitors on cell proliferation, migration, and invasion (Fig. 5d, e).

### LDHA overexpression reverses the anti-tumorigenic effects of LINC01128 knockdown

**Fig. 6:**
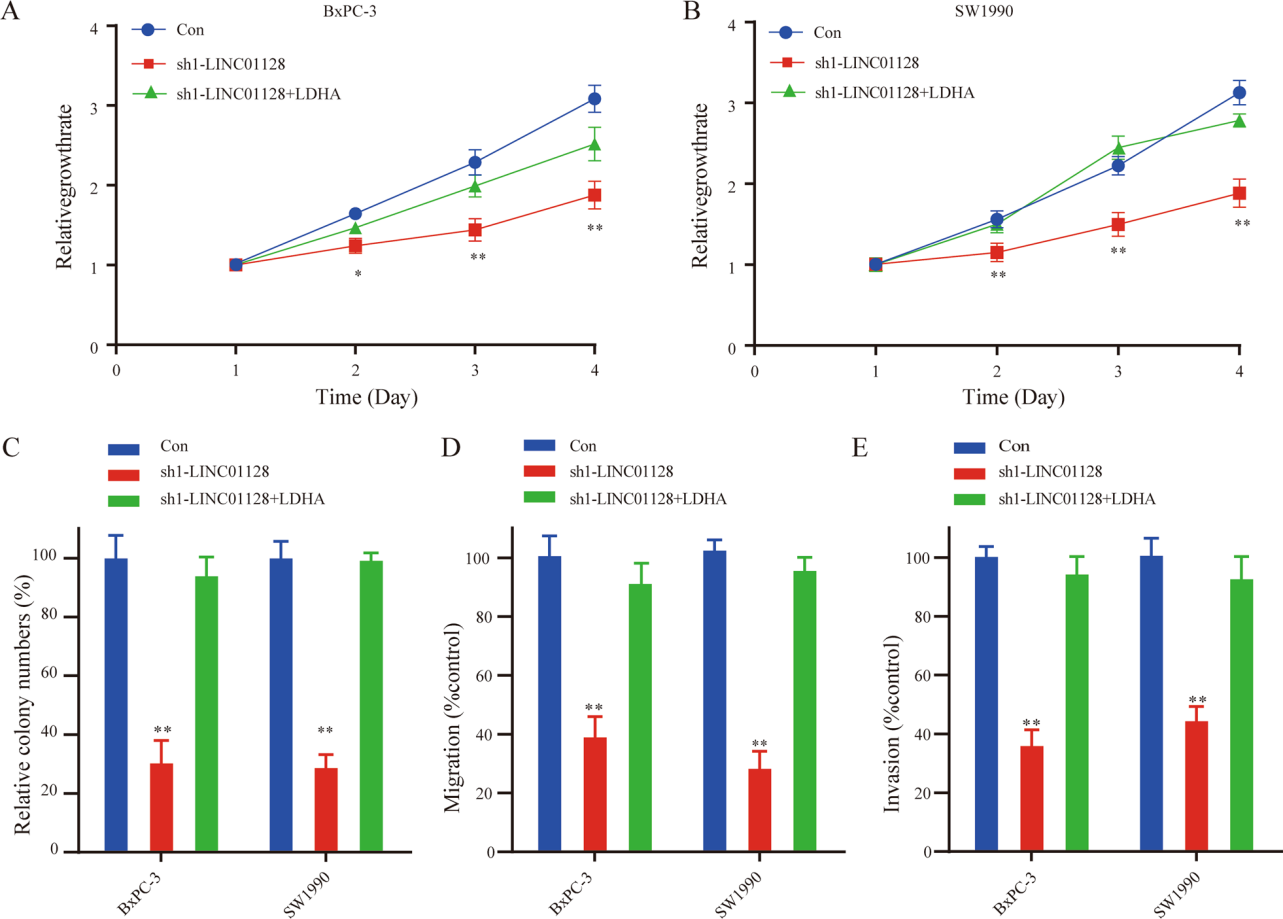
LDHA overexpression activated the tumorigenesis effects of LINC01128 knockdown. **a**, **b** CCK-8 assays were used to determine the proliferation ability of LINC01128 silencing and cells co-transfected with LINC01128 shRNA and LDHA overexpression plasmids. **c** Clone formation assays were used to detect cell viability. **d**, **e** A transwell assay was performed to detect cell migration and invasion abilities. Data are presented as mean ± SD of three independent experiments. *p < 0.05, **p < 0.01

Our previous experiments demonstrated that LINC01128 could promote PC cell proliferation, migration, and invasion by sponging miR-561-5p and that LDHA was the downstream target of miR-561-5p. Thus, we investigated the role of LINC01128 in promoting tumor growth mediating by LDHA. In addition, we overexpressed LDHA in BxPC-3 and SW1990 cells to verified whether LDHA could overturn the anti-cancer effects of sh-LINC01128. The rescue assay showed that LDHA overexpression partially reversed the effects of LINC01128 knockdown on cell proliferation, migration, and invasion in PC (Fig. 6a-e). Taken together, these results indicate that LINC01128 may act as a crucial tumor promoter of PC by competitively binding to miR-561-5p and subsequently upregulating the expression of LDHA.

### LINC01128 promotes tumor growth and regulates LDHA protein expression in vivo

**Fig. 7:**
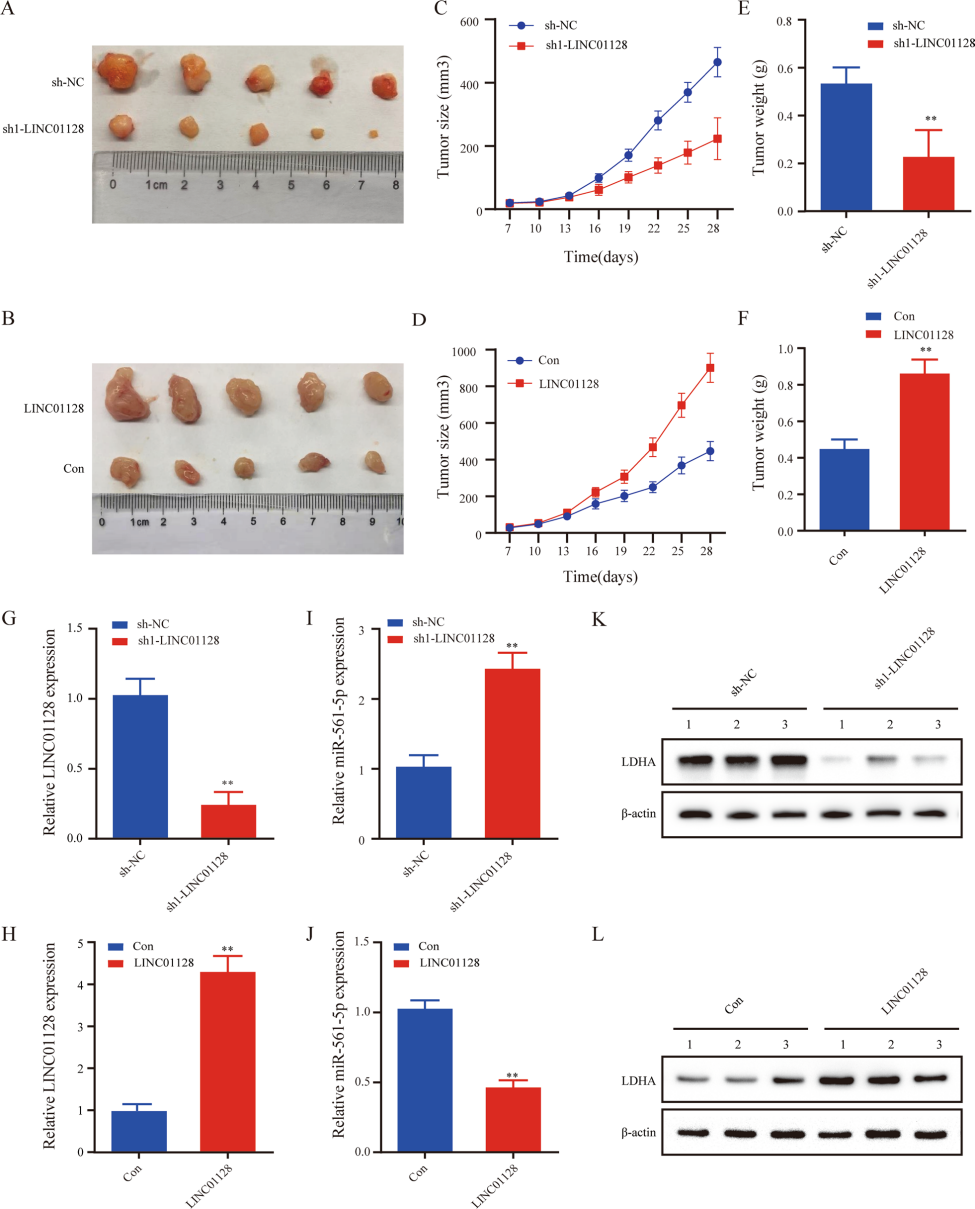
LINC01128 promotes PC tumor growth and protein expression of LDHA in vivo. **a**, **b** An image of a neoplasm in xenograft mouse samples on day 29. **c**, **d** Growth curves of xenograft tumors. **e**, **f** Tumor size and tumor weight of neoplasms. **g**–**j** qRT-PCR was used to determine the expression levels of LINC01128 and miR-561-5p in tumor tissue samples. **k**, **l** Western blot analysis was used to detect the expression of LDHA in tumor tissue samples. Data are expressed as the mean ± SD of three independent experiments. *p < 0.05, **p < 0.01

Previously, we have proved that LINC01128 was overexpressed in PC cells whose knockdown suppressed proliferation, migration, invasion, and EMT of these cells. Next, we performed a xenograft in vivo mouse assay using SW1990 cells and MIAPaCa-2 cells to explore the role of LINC01128 in tumor growth. We obtained tumor images after the mice were euthanized (Fig. 7a, b). The results showed that LINC01128 knockdown decreased tumor size and tumor weight compared to those in the negative control, while LINC01128 overexpression yielded an opposite result (Fig. 7c–f). In the tumor tissue sample, qRT-PCR was used to clarify the transfection efficiency of the sh-LINC01128 and LINC01128 overexpression groups (Fig. 7g, h). An opposite expression level between miR-561-5p and LINC01128 was detected (Fig. 7h, i). Finally, western blot analysis showed that LDHA protein expression was significantly reduced in the sh-LINC01128 group compared to the NC group but mountingly increased in the LINC01128 group (Fig. 7j, k). In conclusion, our results indicate that LINC01128 promotes tumor growth and regulates LDHA protein expression in vivo.

## Discussion

It has been confirmed that lncRNAs are involved in the occurrence and development of various cancers [17], including prostate cancer, bladder cancer, gallbladder cancer, PC, osteosarcoma, and breast cancer [12, 18–21]. Several lncRNAs have been identified, such as THAP9-AS1 [22], PLAT1 [23], PVT1 [24], and XLOC_006390 [25], and their underlying mechanisms have been explored in PC. LINC01128 has previously been implicated in cervical cancer [10] and acute myeloid leukemia [11], however, there have been no reports on its effects in PC. In our study, we found that LINC01128 was upregulated in PC tissues and cell lines, and LINC01128 overexpression was related to the poor prognosis of patients with PC. In addition, LINC01128 knockdown inhibited cell proliferation, migration, invasion, and EMT processes of PC cells and led to G2/M phase arrest, while LINC01128 overexpression yielded opposite results.

Previous studies have shown that lncRNAs can regulate gene expression at the pre-transcriptional level, transcriptional level, and post-transcriptional level according to their locations in the cell [26, 27]. The lncRNAs locating in the nucleus functioned pre-transcriptionally or transcriptionally, whereas lncRNAs located in the cytoplasm usually competing with endogenous RNAs and sponging miRNAs to regulate the expression of target mRNAs post-transcriptionally [28, 29]. LINC01128, a novel lncRNA, has been shown to promote cell proliferation, migration, and invasion, inhibit cell apoptosis, sponge miR-383-5p, thus upregulating the expression of SFN in cervical cancer [10]. However, another study demonstrated that LINC01128 inhibited the malignant behavior of acute myeloid leukemia [11]. These findings may be explained by the fact lncRNAs possess a more pronounced tissue-specific expression pattern compared to protein-encoding genes. Through subcellular grading experiments, we found that LINC01128 was mainly located in the cytoplasm, indicating that LINC01128 may play a role as a ceRNA during initiation and progression by sponging miRNAs. The deregulation of miR-561-5p expression in various cancers indicates that miR-561-5p may play an important role in tumorigenesis and progression [30, 31]. Kun et al. showed that miR-561-5p was frequently down-regulated in gastric cancer cell lines and tissues, and overexpression of miR-561-5p inhibited cell proliferation and invasion by downregulating c-Myc expression [32]. A recent next-generation sequencing study showed that miR-561-5p was also often down-regulated in PC [33]. In our study, StarBase3.0 predicted that miR-561-5p might be a downstream target of LINC01128. Subsequent luciferase reporter and MS2-RIP assays confirmed the interaction between LINC01128 and miR-561-5p. Further experiments have shown that miR-561-5p can promote cell proliferation, migration, and invasion during the initiation and progression of PC, suggesting that the cancer-promoting effect of LINC01128 mainly relies on miR-561-5p.

According to the ceRNA hypothesis, mRNA expression of target genes is upregulated due to the competitive binding of lncRNAs to miRNAs. StarBase3.0 was used to predict LDHA as a potential target gene for LINC01128 and miRNA-561. Previous studies have demonstrated that LDHA is a step-control enzyme in the glycolysis pathway [34, 35]. Increasing evidences have shown that LDHA is upregulated in various cancers, which is related to the clinicopathological characteristics and prognosis of patients and involved in cancer growth [36–38]. In various cancers, including breast cancer, nasopharyngeal cancer, gallbladder cancer, and PC, LDHA can promote malignant progression by increasing the production of lactic acid, accelerating the uptake of glucose, and regulating many cancer-related molecules [39–43]. In a previous study, LDHA silencing induced a decrease in lactic acid concentration, and cancer cell migration was reduced by approximately 40% compared to the control siRNA [44]. In our study, luciferase reporter and MS2-RIP experiments verified the direct binding of LDHA to miR-561-5p. LDHA silencing partially eliminated the tumorigenic effects of LINC01128 in the rescue experiment. Furthermore, LINC01128 promoted tumor growth and regulated LDHA protein expression in vivo. Recently, the application of tumor-related lncRNA HOX Transcript Antisense Intergenic RNA (HOTAIR) deletion mutant variant was defined an innovative RNA-based strategy for tumor therapy reported to reduce cellular motility, invasiveness, growth, and EMT [45]. In addition, Wang et al. [46] also shed great light on the importance of the researches regarding to targeting RNA therapeutics in cancer which aroused widespread attention and interests. Therefore, the development of the novel inhibitor or variant of LINC01128 for PC therapy emerges to be a significant strategy which needs further work in the future.

Taken together, LINC01128 can act as a ceRNA and regulate the expression of LDHA protein by competitively sponging miR-561-5p, thus promoting proliferation, migration, invasion, and EMT of PC cells. This highlights the LINC01128/miR-561-5p/LDHA axis as a potential significantly diagnostic and therapeutic target for patients with PC.

## Conclusions

This study demonstrated that LINC01128 promoted proliferation, migration, invasion, and EMT of PC via regulating the miR-561-5p/LDHA axis, pointing that LINC01128 could act as a novel prognostic predictor and a therapeutic target.

## Supplementary Information

**Additional file 1: Fig. S1.** miR-561-5p exerted an anti-oncogenic biological role in PC cell lines. **a** The relative growth rate of BxPC-3 and SW1990 transfected with miR-561-5p mimics or negative control. **b** The proliferation ability of PC cells with miR-561-5p overexpression. **c-d** The migration and invasion capability change of PC cells after miR-561-5p upregulation. Data are expressed as the mean ± SD of three independent experiments. *p < 0.05, **p < 0.01.

**Additional file 2: Fig. S2.** LINC01128 was correlated positively with LDHA. **a-b** The expression of LDHA detected by qRT-PCR after increasing or decreasing LINC01128. **c-d** Western blotting showed the expression of LDHA upon up- or downregulation of LINC01128. Data are expressed as the mean ± SD of three independent experiments. *p < 0.05, **p < 0.01.

## Data Availability

All the data and materials supporting the conclusions are included in the paper.

## Abbreviations

CCK-8: Cell Counting Kit 8
ceRNA: Competitive endogenous RNA
EMT: Epithelial-mesenchymal transition
LDHA: Lactate dehydrogenase A
lncRNA: Long non-coding RNA
NC: Negative control
PC: Pancreatic cancer
RIP: RNA immunoprecipitation
qRT-PCR: Quantitative reverse transcriptase-polymerase chain reaction
SFN: Stratifin
