# Supplementary figures and images for: LINC01128 facilitates the progression of pancreatic cancer through up-regulation of LDHA by targeting miR-561-5p
Source: Cancer Cell Int. 2022 Feb 22;22:93. doi: 10.1186/s12935-022-02490-5 (PMC8862213; doi:10.1186/s12935-022-02490-5)

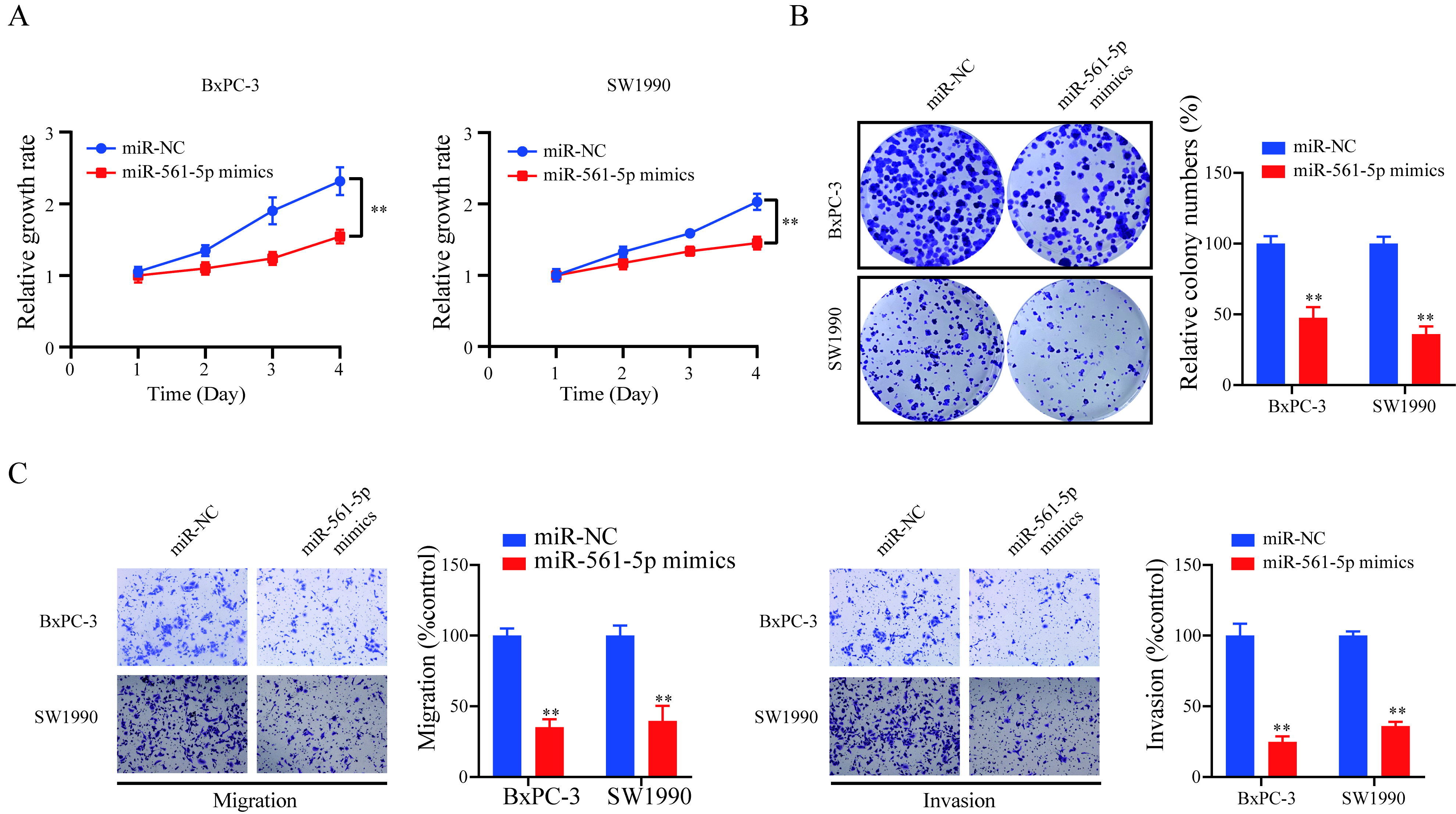

Supplement: Supplementary file 1 — Additional file 1: Fig. S1. miR-561-5p exerted an anti-oncogenic biological role in PC cell lines. a The relative growth rate of BxPC-3 and SW1990 transfected with miR-561-5p mimics or negative control. b The proliferation ability of PC cells with miR-561-5p overexpression. c-d The migration and invasion capability change of PC cells after miR-561-5p upregulation. Data are expressed as the mean ± SD of three independent experiments. *p < 0.05, **p < 0.01. [file 12935_2022_2490_MOESM1_ESM.tif]

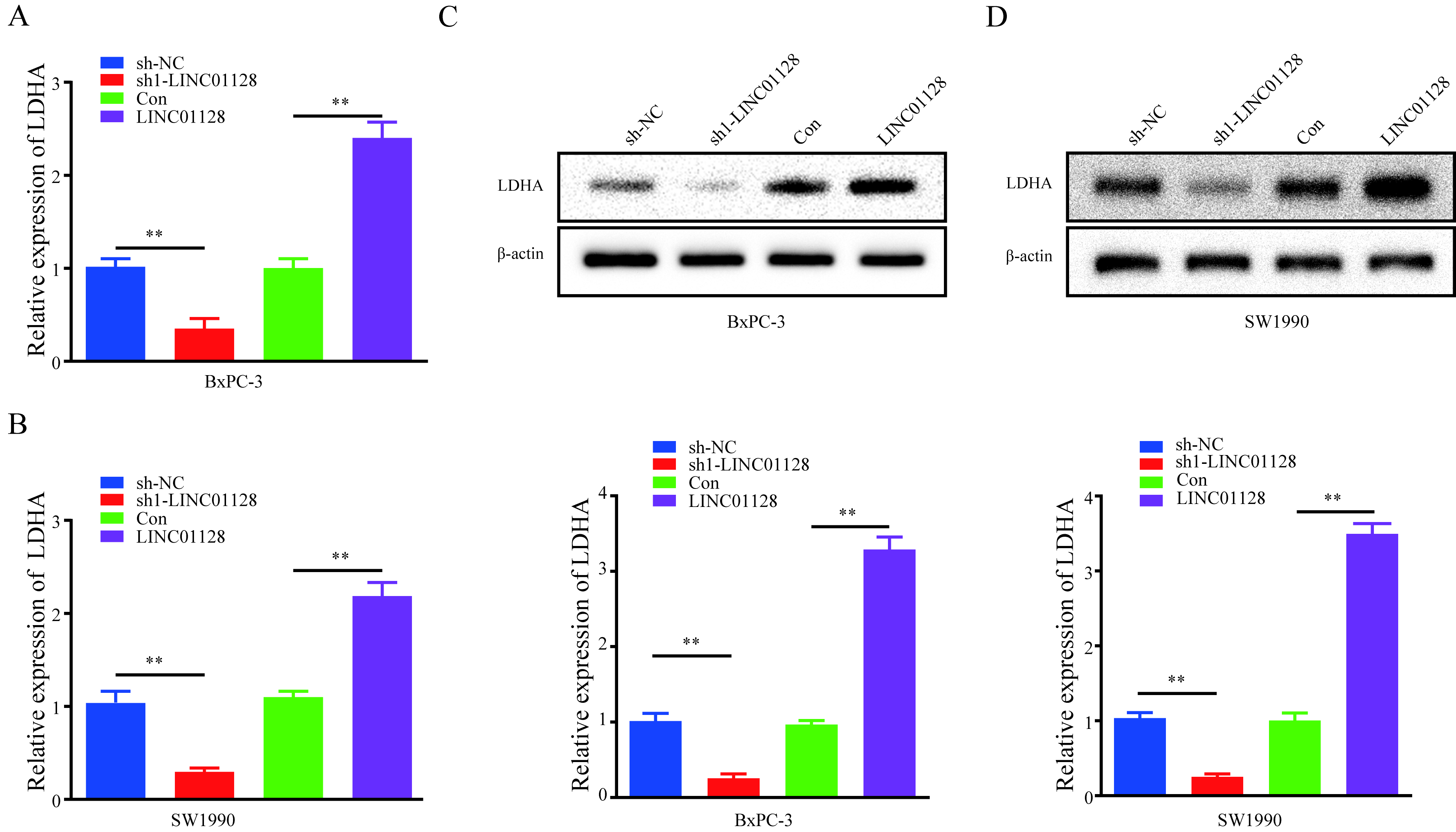

Supplement: Supplementary file 2 — Additional file 2: Fig. S2. LINC01128 was correlated positively with LDHA. a-b The expression of LDHA detected by qRT-PCR after increasing or decreasing LINC01128. c-d Western blotting showed the expression of LDHA upon up- or downregulation of LINC01128. Data are expressed as the mean ± SD of three independent experiments. *p < 0.05, **p < 0.01. [file 12935_2022_2490_MOESM2_ESM.tif]
